# Refuges from fire maintain pollinator–plant interaction networks

**DOI:** 10.1002/ece3.5161

**Published:** 2019-04-30

**Authors:** Opeyemi Adedoja, Carsten F. Dormann, Temitope Kehinde, Michael J. Samways

**Affiliations:** ^1^ Department of Conservation Ecology and Entomology Stellenbosch University Stellenbosch South Africa; ^2^ Biometry and Environmental System Analysis University of Freiburg Freiburg Germany; ^3^ Department of Zoology Obafemi Awolowo University Ile‐Ife Nigeria

**Keywords:** fire, flowering plants, flower‐visiting insects, harsh conditions, refuge, specialization

## Abstract

Fire is a major disturbance factor in many terrestrial ecosystems, leading to landscape transformation in fire‐prone areas. Species in mutualistic interactions are often highly sensitive to disturbances like fire events, but the degree and complexity of their responses are unclear. We use bipartite insect–flower interaction networks across a recently burned landscape to explore how plant–pollinator interaction networks respond to a recent major fire event at the landscape level, and where fire refuges were present. We also investigate the effectiveness of these refuges at different elevations (valley to hilltop) for the conservation of displaced flower‐visiting insects during fire events. Then, we explore how the degree of specialization of flower‐visiting insects changes across habitats with different levels of fire impact. We did this in natural areas in the Greater Cape Floristic Region (GCFR) biodiversity hotspot, which is species rich in plants and pollinators. Bees and beetles were the most frequent pollinators in interactions, followed by wasps and flies. Highest interaction activity was in the fire refuges and least in burned areas. Interactions also tracked flower abundance, which was highest in fire refuges in the valley and lowest in burned areas. Interactions consisted mostly of specialized flower visitors, especially in refuge areas. The interaction network and species specialization were lowest in burned areas. However, species common to at least two fire classes showed no significant difference in species specialization. We conclude that flower‐rich fire refuges sustain plant–pollinator interactions, especially those involving specialized species, in fire‐disturbed landscape. This may be an important shelter for specialized pollinator species at the time that the burned landscape goes through regrowth and succession as part of ecosystem recovery process after a major fire event.

## 1 INTRODUCTION

Fire is a major disturbance factor in many terrestrial ecosystems (New, 2014). It is especially prevalent through the recent increase in human‐induced landscape transformation and rapid climate change, especially in Mediterranean‐type ecosystems, where warmer and drier conditions are increasingly prevalent (Archibald, Staver, & Levin, 2012; Bowman et al., 2011; Steel, Safford, & Viers, 2015; Syphard, Radeloff, Hawbaker, & Stewart, 2009). The immediate impact of fire usually results in high mortality of resident species, which increases with intensity and frequency of the fire (Adeney, Ginsberg, Russell, & Kinnaird, 2006; Bennett et al., 2016; Silveira, Barlow, Louzada, & Moutinho, 2010). Flower‐visiting insects, especially the less mobile species, are greatly affected by fire in natural landscapes. However, species functional traits may influence survival during fire, for example, while zoophagous and phytophagous arthropods are highly resilient to the effects of fire, mortality was higher for ground‐litter saprophagous species (Moretti, Bello, Roberts, & Potts, 2009). Also, specialist bee species decline more than generalists in freshly burned habitat (Peralta, Stevani, Chacoff, Dorado, & Vázquez, 2017). In addition, long‐term recolonization of burned habitat may be affected by transformation processes of the habitat, as the newly transformed habitat may yield different species composition. Over the postfire period, fire usually transforms landscapes into more open habitat, which may change species composition over time (Case & Staver, 2017). This is seen in South Africa, where the composition of the butterfly assemblage changed over the period of recovery following a major fire event (Pryke & Samways, 2009; Yekwayo, Pryke, Gaigher, & Samways, 2018).

Most studies on fire show a positive influence of fire on flowering plant diversity and abundance of insect pollinators (Bond & Scott, 2010; Lamont & Downes, 2011; Ponisio et al., 2016). While this is important for the long‐term biodiversity succession in fire‐disturbed ecosystems, there is concern for the immediate species response during and after fire. Potts et al. (2003) showed a time lag of 2 years for burned area to reach full recovery and a flowering peak. Immediately after fire, a decline in pollinator abundance and floral resources is expected in burned areas, yielding a temporal decline in plant–pollinator interactions. During this time, while flowering plants are burned down, mobile insect pollinators seek refuge in areas not impacted by fire. Refuges are areas in an ecosystem where a disturbance affecting a larger region did not take place. As a consequence, they can buffer the effect of transformation events in natural landscapes (Mackey, Lindenmayer, Gill, McCarthy, & Lindesay, 2002). Despite the great importance of refuges on the recovery process and resilience of populations, they are rarely studied (Robinson et al., 2013). In fire‐prone areas, patches of vegetation that escape the full impact of fire can serve as refuges for individuals of certain insect species (Bradstock, Bedward, Gill, & Cohn, 2005; Burton, Parisien, Hicke, Hall, & Freeburn, 2008; Castro, Moreno‐Rueda, & Hódar, 2010; Perera, Nuse, & Routledge, 2007). However, for a patch to function effectively as a refuge, it must provide enough floral and nesting resources for survival of the locally lost or displaced flower‐visiting insect species (Brown, York, Christie, & McCarthy, 2017; Watson et al., 2012). As fire ultimately leads to temporal displacement of flower‐visiting insects during the fire event, refuge patches are essential for local survival and even persistence of flower‐visiting insects.

Insect pollinators forage in areas close to their nest (Gathmann & Tscharntke, 2002; Schweitzer, Capuano, Young, & Colla, 2012). The location of important fire refuges in the disturbed landscape may be important for the persistence of specific pollinator species in fire‐disturbed landscape. While some large bees can visit vegetation patches for floral resources over long distances, ground‐dwelling and less mobile groups may require nesting resources within patches around the burned area (Steffan‐Dewenter, 2002). In addition to site‐specific abiotic components, such as nutrient availability, canopy cover may influence the effectiveness of fire refuges and play a significant role in the conservation of insect pollinators during fire disturbance. For example, changes across elevation may influence flowering plant distribution, with plants at higher elevations having reduced growth (Boscutti et al., 2018) and low species richness (Jacquemyn, Micheneau, Roberts, & Pailler, 2005).

Most times, pollinators are displaced from areas of few flowers at high elevations to flower‐rich lower elevations (Adedoja, Kehinde, & Samways, 2018). Unlike hilltops, valleys sometimes riparian corridors with rich vegetation, and which are essential for effective nest provision for insect pollinators, especially bees (Mader, Shepherd, Vaughan, Hoffman Black, & Lebuhn, 2011). In the context of fire refuges, it is expected that areas of sufficient requirements for nesting will make better refuges during fire disturbance. However, there is little information on the effectiveness of fire refuges across heterogeneous topographic landscapes.

Network metrics are used to describe the properties of interaction networks. Most of the metrics are standardized ways of explaining the contribution of individual species and communities in a network leading to the success of interactions and delivery of ecosystem functions. For example, there is a simple approach to estimating species specialization in a network where it is possible to directly link a species to all interacting partners by observation (Johnson & Steiner, 2003; Ollerton, Killick, Lamborn, Watts, & Whiston, 2007). However, this approach is limited by not taking into account the estimates of resource diversity. A more inclusive index for species specialization is index d’, which takes into account the diversity of interacting partners and their importance in a network based on observed and expected interacting frequencies (Blüthgen, Menzel, & Blüthgen, 2006). By using this index, species that interact with more partners, in relation to their importance in a network, are more generalized compared to specialized species that are more sensitive to random selection of interacting partners in a network. Overall, network metrics can be used to explore community structure, especially for mutualistic species. While abundance and distribution of interacting partners may be relatively stable in less disturbed areas, fire disturbance in fire‐prone landscapes may facilitate fluctuations in species abundance and distribution. Over time, more generalized species become increasingly abundant in areas with frequent fires with short‐term intervals (Peralta et al., 2017). As a consequence, the interactions consisting of more specialized species in burned areas may face a breakdown (Forrest, 2015; Memmott, Craze, Waser, & Price, 2007). For example, a specialist pollinator may be forced to explore other available floral resources in a smaller refuge patch when it is displaced from its extensive habitat.

Despite the high impact of fire in changing natural landscapes and community interactions, there is little information on the response of plant–pollinator interactions to fire events, and how fire refuges alter species response to fire. Here, we explore how plant–pollinator interaction networks respond to recent fire at the landscape level where fire refuges are present. We also investigate the effectiveness of these refuges at different elevations for the conservation of displaced flower‐visiting insects during fire events. Refuges have an important relationship with elevation and rugosity of landscape as these features contribute to the leaving of areas that avoid being burned.

We undertake this study in the flower‐ and pollinator‐rich Greater Cape Floristic Region (GCFR) biodiversity hotspot. We also explore how the degree of specialization of flower‐visiting insects changes across habitats with different levels of fire impact. To this end, we compile information from observations on bipartite insect–flower interaction networks from visitation to flowers by important flower‐visiting insects across a recently burned landscape. We hypothesize that (a) like most disturbance events, the direct impact of fire is expected to aid species displacement to a less disturbed area, thereby we expect that unburned natural areas will have highest abundance of flowering plants and highest interaction frequency compared to burned and refuge habitats; (b) geographical valleys are often nutrient rich with streams running along them, and so have more flowering plant species that may act as refuges in the valley, and are therefore more effective in sustaining interactions compared to those on hilltops, and (c) more flower‐interacting partners in unburned habitat will influence a more specialized networks compared to those in refuges and burned areas.

## 2 MATERIALS AND METHODS

The study was conducted in the large natural set‐aside areas on wine farms in the Western Cape Province, South Africa, in the Greater Cape Floristic Region (GCFR) biodiversity hotspot. Bee diversity in the GCFR is exceptionally high, coinciding with that of plants (Kuhlmann, 2005). Two adjacent wine estates were selected (Vergelegen: 34.0764°S, 18.8899°E and Lourensford: 34.0719°S, 18.8886°E). These estates practice biodiversity‐friendly agriculture, with extensive areas of the farms devoted to conservation of indigenous biodiversity, where our sites were positioned. The landscape varies in topography, with sites available in valleys, on hill slopes, and on hilltops. These natural areas on the estates burned, but left refuges December 2016–February 2017.

We classified our sampling sites into those in refuges (2 years since last fire), burned (6 months since last fire), and unburned areas (9–10 years since last fire). Refuge sites were defined as patches ≥50 m^2^ and of unburned vegetation within the burned matrix. Unburned sites were those in extensive natural areas that were beyond the fire front. Sites were selected in the valleys (≤200 m a.s.l.), on hill slopes (180–250 m a.s.l.), and on hilltops (400–450 m a.s.l.). For every valley site, we also sampled matching hillslope and hilltop sites. Plant–pollinator interactions were recorded at 27 sites across the fire categories late August–November 2017.

A total of nine sites, each of 50 m^2^, were in each of the refuges, burned areas, and also unburned areas (i.e., 9 sites × 3 fire classes = 27). Burned and refuge sites were selected in pairs ≥ 100 m apart from the edge, which in turn, were 0.9–3 km from the unburned sites. For every burned site, we selected the closest refuge patch that matched the size of a study site (i.e., 50 m^2^) in each elevation category. The refuge and burned sites in each elevation category were visited on the same sampling day, and observation time was altered between fire class in the second visit.

Timed observation of insect activity was standardized to 10 min/2 m^2 ^plot to avoid overemphasizing the specialization of flowering plants (Gibson, Knott, Eberlein, & Memmott, 2011), reducing sampling bias from variables such as flower abundance. During this time, an interaction was noted when an insect visited the floral unit of a plant.

Flower‐visiting insects were identified in the field or caught for later identification as morphospecies. Five replicates per 2 m^2^ sampling unit within each site yielded a total of 50 min observation time per site per sampling period. Every site was visited twice, with a total of 100 min observations per site, which were pooled as a single interaction network. Flower abundance of each plant species was estimated in each 2 m^2^ plot where insect activities were observed. A flower unit is defined here as the unit from which a honeybee‐sized insect will fly to the next unit rather than crawl (Dicks, Corbet, & Pywell, 2002).

We also estimated flower area of display for each flowering plant species. Area of floral display was determined for each open flowering plant species by measuring the diameter of 1–10 flowers per plant species. Areas of flowers with circular outline were estimated using π*r* ^2^ and L × B for those flowers with a more rectangular surface outline. A flower with visible depth, such as that of *Protea repens*, was estimated using 2 π*r* ^2^ *d* + π*r* ^2^. The mean flower area for a plant species, together with the total abundance of flowers, was used to estimate the plant flower area per site.

### 2.1 Statistical analyses

Interaction matrices for plant–pollinator interaction networks were compiled for each site. Data were analyzed using the bipartite package in R (Dormann et al., 2008). Network‐ and species‐level indices were computed for each of the 27 networks: connectance, weighted nestedness (NODF), network specialization (H_2_′), normalized degree (ND), and species specialization (*d*′). Network and species specialization indices were selected for this purpose, as these are insensitive to diversity of interacting partners (Schleuning et al., 2012), and so are suitable for this kind of study where effect of fire is expected to influence flowering plant diversity across our study sites.

To account for biases in estimates of interaction metrics, especially specialization which could result from differences in activities or attractiveness of interacting partners, we employed null models for the quantitative network metrics, based on the observed number of interactions for a species in a given network (using the Patefield algorithm: Dormann, Fründ, Blüthgen, & Gruber, 2009). We computed 100 null models for each network and calculated z‐scores for each network metric (i.e., differences between observed and mean null model index values, divided by the standard deviation of the null model values). The application of null models here reduces the biases in estimating network indices, especially with differences in number of interactions across our study sites.

To assess the differences in interaction frequency, species richness, abundance of flowering plants, and flower area across fire classes, and elevation, we used a generalized mixed‐effect models. We specified sites as random variables to account for possible overlap among the study sites. Fire classes and elevation were the explanatory variables in our model. We also assessed the interaction among explanatory variables in our model. Similarly, we assessed how flower abundance and area of display influenced the pattern of interaction frequency across elevation and fire class using a generalized linear model. Also, to understand how z‐scores of network metrics change across elevation and fire classes, we used GLMs, with fire class and elevation as predictors. We computed a PERMANOVA to analyze the difference in species composition of pollinators observed in interaction among fire class and elevation. Analyses were carried out using the packages lme4 and vegan.

To understand how interactions of pollinators are structured by availability of interacting partner across fire classes, we used the normalized degree function (ND) in the bipartite package. ND shows the degree of generalization of pollinator species through the sum of links scaled by the number of possible partners for individual species in a network. Here, we computed the relationship between interaction frequency and the ND of each species, and we observed how this changed across fire class. We used a generalized mixed‐effect model for this purpose. We specified species as a random factor, to assess the confounding effect of different ND and interaction values of the same species in different fire class.

Finally, to understand the degree of specialization of individual species in the network across fire class, we computed each species’ *d*′‐value. Species specialization index (*d*′) was used to measure the degree of discrimination of a species from random selection of partners in a network. Index d’ is constructed in such a way that it measures specialization not in absolute terms but relative to the other pollinators and resource abundances (Blüthgen et al., 2006). We assessed how observed *d*′‐values changed across fire class and elevation using a linear mixed‐effect model, specifying species as a random factor. We also used the z‐scores of *d*′‐value from the null model following the same approach. Then, to assess whether species change the degree of specialization in different fire classes, we selected species common to at least two fire classes, and we assessed how their *d*′‐values changed across fire class.

## 3 RESULTS

**Figure 1:**
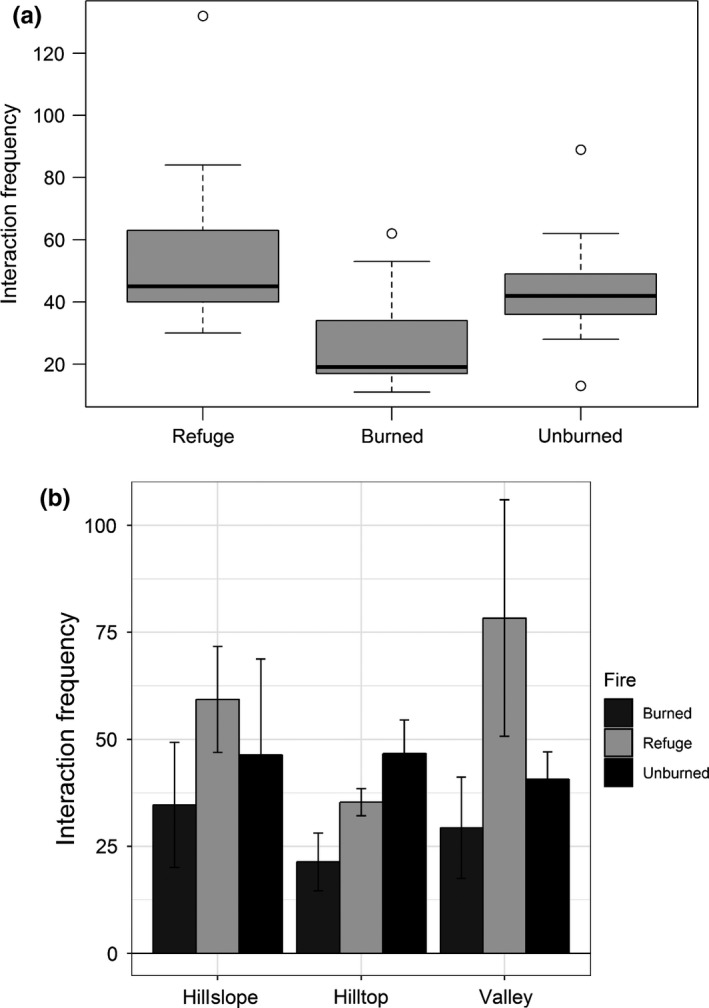
Mean interaction frequency (±*SE*) among (a) fire classes and (b) fire classes (averaged across elevations) and elevation (averaged across fire classes)

A total of 1,176 interactions were recorded among 67 insect (Appendix S1) and 56 plant species (Appendix S2). Interactions consisted of bees (55.6%), beetles (25.94%), flies (17.09%), and wasps (1.53%). There was a significant difference in interaction frequency among fire classes. Highest interaction was observed in fire refuges which was significantly different from the lowest interactions observed in burned areas (Z = 4.837, *p* < 0.001, Figure 1a). There was no signification difference in interaction frequency across elevation. However, we found a significant interaction between the explanatory variables (elevation and fire class) on pollinator interaction frequency (χ^2^ = 20.236, *p* < 0.001, Figure 1b). While interaction was highest in the refuges at the valley and hillslope sites, interaction was highest in unburned sites at the hilltop.

**Figure 2:**
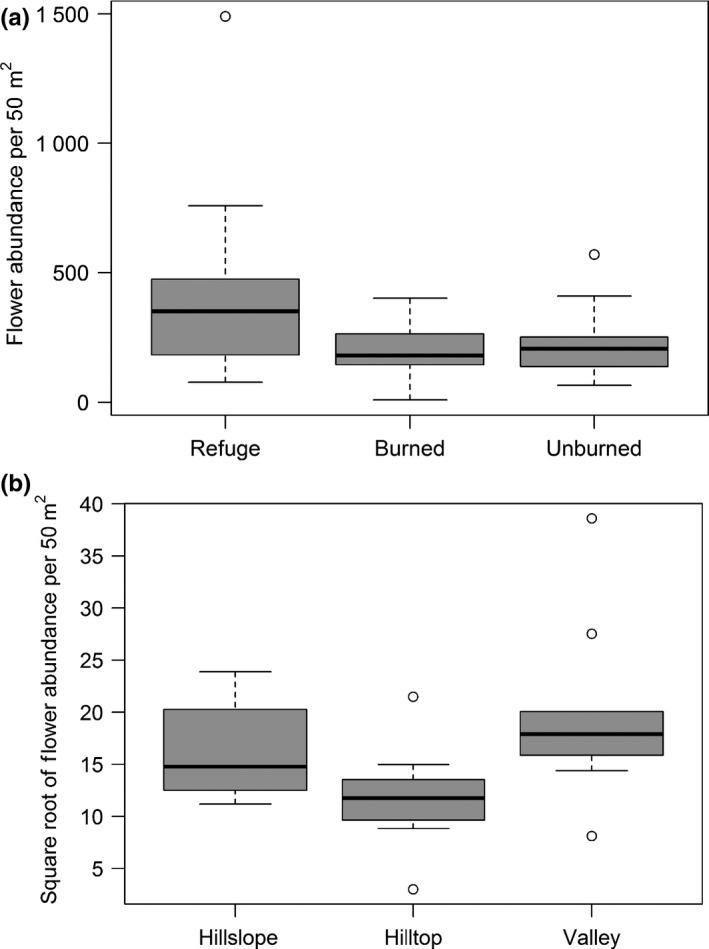
Mean flower abundance (±*SE*) across (a) fire classes and (b) elevation category

There was a significant difference in flower abundance across fire classes. Highest flower abundance was observed in fire refuges, while lowest in burned areas (Z = 2.825, *p* < 0.01, Figure 2a). Also, there was a significant difference in flower abundance across elevation. Flower abundance was highest in the valley and lowest on the hilltop (Z = 2.118, *p* < 0.05, Figure 2b). However, there was no significant difference in flower area of display across fire class or elevation. Also, there was no significant difference in flowering plant and pollinator species richness across fire class or elevation.

There was no significant difference in species composition of pollinator assemblages across fire class (*F*_2, 24_ = 1.0668, *p* = 0.347) or elevation (*F*_2, 24_ = 1.1123, *p* = 0.273). Also, there was no significant difference in flowering plant composition across elevation (*F*_2, 24_ = 1.1163, *p* = 0.211). However, there was a significant difference in species composition of flowering plants across fire class (*F*_2, 24_ = 1.4611, *p* < 0.01).

Overall, flower abundance (*F* = 85.92, *p* < 0.001) and flower area (*F* = 13.14, *p* < 0.001) significantly influenced pollinator activities. However, while flower abundance significantly influences pollinator activity across topography (*F* = 5.79, *p* < 0.01), there was no significant influence of flower abundance on pollinator activities among fire classes (*F* = 1.08, *p* = 0.34). On the other hand, flower area significantly influences pollinator activity across fire class (*F* = 12.07, *p* < 0.001). However, there was no significant influence of flower area on pollinator activity across topography (*F* = 1.27, *p* = 0.28).

**Figure 3:**
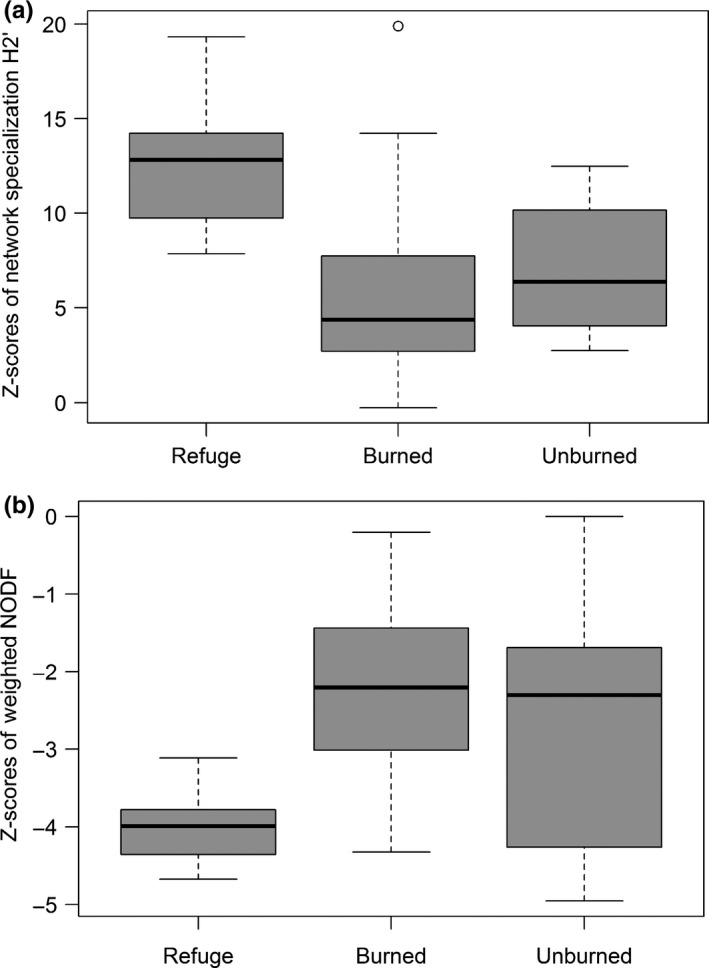
Mean z‐scores (±*SE*) of (a) network specialization (H_2_′) and (b) weighted nestedness (NODF) across fire classes

The average network specialization (H_2_′) value across the 27 study sites was high (mean = 0.736, standard deviation = 0.214). There was a significant difference in z‐scores of network specialization (H_2_′) among fire classes (*F*_2, 24_ = 4.30, *p* = 0.025). Highest network specialization was at refuge sites, and this was significantly different from the lowest H_2_′ at the burned sites (Figure 3a). However, H_2_′ was not significantly different across elevation. Weighted nestedness (NODF) also differed significantly among the fire classes (*F*_2, 24_ = 5.581, *p* = 0.01). Networks at refuge sites were less nested than those at burned sites (Figure 3b). However, there were no significant differences in NODF across elevation. There were no significant differences in network connectance across fire and elevation classes (*p* > 0.05).

### 3.1 Species‐level specialization

Overall mean of per‐flower‐visiting species *d*′ (mean = 0.407, *SD* = 0.323) indicates that the flower‐visiting insects were moderately specialized. Flowering plants, on the other hand, were highly specialized with overall mean per‐species *d*′ (mean = 0.972, *SD* = 0.167). There was no significant difference in *d*′‐value across fire classes (*F* = 2.913, *p* = 0.0565). However, after correction by the null model, we found a significant difference in d’‐value across fire classes (*F* = 7.123, *p* = 0.001). Highest z‐scores for *d*′‐values were observed at the refuge sites and lowest at the burned sites.

When we compared, for common flower‐visiting insects, their specialization in the three fire classes, we found no significant differences among fire classes (*F* = 1.983, *p* = 0.145) or elevation category (*F* = 1.083, *p* = 0.344).

**Figure 4:**
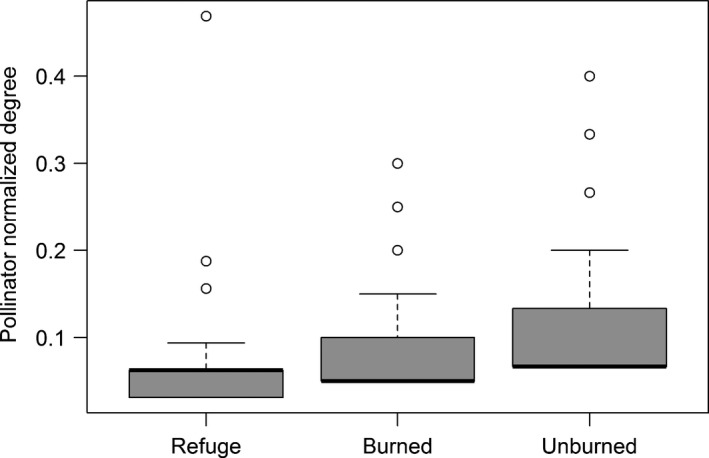
Mean normalized degree (ND) (±*SE*) across fire classes

There was a significant difference in pollinator normalized degree (ND) across fire class (*F* = 29.89, *p* < 0.001). ND was highest in unburned sites, followed by burned sites, and lowest at refuge sites (Figure 4). There was also a significant relationship between species interaction and generalization across fire classes (*F* = 24.357, *p* < 0.00001). ND values were highest for pollinators involved in interactions at unburned sites and lowest for interactions at refuge sites (Z = −2.202, *p* = 0.0277).

## 4 DISCUSSION

The influence of fire across the landscape is usually uneven. Remnant patches are left behind after fire, creating a mosaic of biodiversity. We found that fire refuges had the highest flower resources and plant–pollinator interactions, compared to the recently burned areas, and also compared to the unburned areas beyond the fire front.

Overall, abundant floral resources, especially for mass flowering plants, were important for the high species interaction observed in the refuges. While generalization (quantified as normalized degree, ND) was high in unburned and burned sites, more specialized species were involved in interactions in fire refuges. This is surprising, as one would have expected a less discriminating behavior as more individuals visit flowers (“scramble competition”), compared to the lower‐density unburned sites. However, species response to environmental stress such as fire is apparently complex. This may be explained by several factors, which we now discuss.

### 4.1 Interaction frequency and species abundance

Fire can impact plant–pollinator interactions in several ways, most of which hinge on resource availability in fire‐prone landscapes (Brown et al., 2017). For a site to be an effective refuge, there must be sufficient nesting and floral resources for the survival and persistence of flower‐visiting insects (Robinson et al., 2013). While high flower abundance drives insect activities across elevation, surface area of flowers influences insect visitation activities across fire classes, as seen here.

Although flower abundance plays a significant role in pollinator visitation, mass flowering, which is an essential feature of most flowering plant species of the GCFR, increases pollinator activity in this highly diverse hotspot (Simaika, Samways, & Vrdoljak, 2018; Vrodjilak, Samways, & Simaika, 2016). In our study, flower abundance was highest in the refuges and lowest at burned and unburned sites. The difference between refuge (two years of fire history) and unburned areas (9–10 years fire interval) in our study is consistent with most studies on the impact of fire on flowering plant distribution. For example, Mola and Williams (2018) found a more prolonged time of interaction in recently burned areas where floral abundance persisted for a longer period of time compared to the unburned places. Also, Campbell, Hanula, and Waldrop (2007) illustrate how pollinator abundance and richness increases with reduced canopy of natural areas and increased understory vegetation. Here, we found that the unburned habitat with fire interval of ten years is overgrown with more of shrubs, weeds, and less visible flower units. However, not only did the refuges not have enough time to regenerate, these areas are relatively open, with fewer shrubs and without tree canopy, unlike the unburned areas. Flowering plants on Mt Carmel national reserve in Israel reached peak flowering two years after fire, so increasing pollinator diversity. However, this peak steadily declined over the next 50 years (Potts et al., 2003). This emphasizes the importance of flower‐rich open habitat in the conservation of flower‐visiting insects and their important role in ecological interactions (Carvalheiro et al., 2011; Holzschuh, Steffan‐Dewenter, Kleijn, & Tscharntke, 2007; Vrdoljak et al., 2016).

The difference between the burned and the refuge areas in terms of flower resource abundance may in turn also be linked to the time taken for resource redistribution in this area. The burned habitat was sampled six months after the fire incidence, when most flowering plants here at this time are at an early emerging period. Full flower regeneration is essential for the visitation of required pollinator in fire‐impacted habitat (Potts et al., 2003). This is the key driver of low flower abundance and interaction in the burned area compared to the flower‐rich refuge habitats. Overall, this shows the importance of nearby rich refuges, where insect pollinators can seek floral requirements, until full regeneration of the burned habitat following fire disturbance.

### 4.2 Network and species specialization

Our results showed high network specialization in refuges in comparison with unburned and burned sites. Similarly, more specialized species were present in interactions in the refuge networks compared to unburned and burned networks. Also, species in two or more fire classes had similar *d*′‐values, implying fire class did not alter species specialization behavior in their response to the changes caused by fire. This means that specialized species then can remain associated with the most preferred flowers at sites with high flower abundance. However, at sites with limited floral resources, flower‐visiting insect species cannot afford to be selective in seeking their most preferred flowers. This pattern was also observed by Peralta et al. (2017), where fewer specialist species were found in burned sites with low flower abundance. Similarly, Plowman et al. (2017) recorded a breakdown in interaction networks and reduced network specialization with a decrease in interacting partners. In our study, normalized degree, which explains species ability to establish links with multiple interacting partners, was highest at unburned and burned sites. This is also supported by high ND values for species in interactions at unburned and burned sites compared to fire refuges.

These findings emphasize the importance of fire refuges as a shelter for displaced pollinators, especially the specialized species with limited range of floral resources. Furthermore, resource availability plays a crucial role in the persistence of specialized species at refuge sites. This implies that it is essential for refuges to be rich in required flowering plant species necessary for interacting insect species, especially specialists, while the burned area recovers from the effects of fire.

Species composition of insects involved in interactions was not significantly different among fire classes. Although we could not assess community composition prefire across study sites, the largely similar insect species composition involved in interactions across fire classes supports the possibility of movement of insects among burned, refuge, and unburned sites. While ground‐dwelling pollinators will find burned habitat most suitable as a result of less ground cover, species here are likely to use power flight to reach other sites (refuges and unburned) in search of suitable floral resources. This is expected to influence the pollinator network and species specialization across fire classes.

Overall, mean network specialization (H_2_′) was high in our study area, supporting high community specialization of plant–pollinator interaction networks in the GCFR (Pauw & Stanway, 2015). However, the low species specialization (*d*′) in our study may be linked to depleted resources resulting from fire disturbance. Since H_2_′ and *d*′ values are linked in interaction networks (Blüthgen et al., 2006), the high network specialization observed here can be influenced by the high *d*′ value for flowering plants compared to lesser mean *d*′ value of flower‐visiting insects. This also supports Pauw and Stanway (2015), where higher *d*′ values were recorded for flowering plants in this region compared to visiting pollinators. Although overall we recorded few interactions, especially in burned areas, the *d*′ and H_2_′ metrics are insensitive to sampling efforts and diversity of interacting partners (Schleuning et al., 2012).

The difference in species specialization (*d*′) among fire class may be attributed to competition among flower‐visiting insect species, especially in habitat with low or few flower resources. The burned sites here were sampled 6 months after fire incidence, and this area had the first set of resprouting flowering plants, but in low abundance compared to refuges. This would increase competition of resident insect pollinators in this area for the scanty resources. Exclusivity of interactions among individual species is more prominent in habitats with more interacting partners, yielding higher species specialization (*d*′) values (Pauw & Stanway, 2015) than were seen here.

Globally, a trend of higher specialization in the species‐rich tropics has been reported with a decline toward temperate areas (Dalsgaard et al., 2009; Dyer et al., 2007; Pauw, 2013). This matches the limited resources in the burned areas here influencing the less specialized species. This means that over time, with less resources at the burned sites, the refuges may serve as an alternative for more specialized species until burned sites regrow with more floral resources.

Species specialization (*d*′) values for common species across the fire classes were not significantly different. This suggests that species remain in a specialized relationship with associated flowers across all fire classes. Flower‐visiting insects, especially solitary bees, find floral resources in areas close to their nesting sites (Gathmann & Tscharntke, 2002). This could also be influenced by mobility of the various insect species, with large bees foraging > 3 km, while small solitary bees seek floral resources < 500 m of their nesting sites (Steffan‐Dewenter, 2002; Steffan‐Dewenter & Tscharntke, 1999). Since we found no significant differences in flowering plant species richness across fire categories, this means that specialized insect species find their preferred flowers within their maximum flight distance.

### 4.3 Interaction network nestedness and species distribution

We found networks to be more nested at burned sites and least at refuge sites. Unlike H_2_′, where interacting species can be selective and retain unique partners, nestedness showed that generalists and specialists in our interaction networks share similar resources (Spiesman & Inouye, 2013). In habitats with high network nestedness, poorly linked and rare species are able to secure interaction partners, as these are linked to more stable components of the network (Bascompte, Jordano, Melián, & Olesen, 2003; Gibson, Nelson, Hopkins, Hamlett, & Memmott, 2006; Memmott, Waser, & Price, 2004). Although, it is difficult to interpret nestedness in small networks (Olesen, Bascompte, Dupont, & Jordano, 2007), our null models nevertheless corrected for this effect. The more nested networks at the burned sites, especially those located on hilltops, may be linked to low flower abundance and less resources for flower‐visiting insects. This increases the opportunity for generalist and specialist species to interact with the same flower partner in the network. This also suggests that the presence of flowering plant species is able to maintain such interactions with insect mutualists in burned and less favorable habitat. Indeed, well‐linked drought‐resistant plant species are important to community resilience and network persistence during harsh conditions in the environment (Lance, Bailey, Lindsay, & Cobb, 2017).

## 5 CONCLUSIONS

Reducing biodiversity loss involves understanding how different components of natural landscapes can be optimized for the conservation of biodiversity and ecological processes during transformation events. Refuges can be part of this loss reduction, with our fire refuges being, in effect, temporary holding areas into which the flower‐visiting insects can retreat while the burned matrix goes through regrowth and succession as part of natural ecosystem recovery. This is likely to be a process that has been honed for many millennia in fire‐prone systems such as the GCFR. It is also promoted by the cragginess of the topography in this system, which provides natural fire refuge areas. Conservation of flower‐visiting insects, along with much other biodiversity (Pryke & Samways, 2012a,2012b; Yekwayo et al., 2018), appears to be naturally adapted to these retreats from fire, enabling populations to survive in patches even when much of the area burns. In turn, it is conceivable in evolutionary terms that this has not only contributed to the generation of high flower diversity in the area, but also that of their insect mutualists.

## AUTHORS CONTRIBUTION

OA, MJS, and TK designed the study, and OA conducted the fieldwork. OA and CFD conducted the statistical analysis and data interpretation. OA wrote the first draft of the manuscript; all authors commented and contributed to the writing of the manuscript.

## Data Availability

Data available from Dryad Digital Repository https://doi.org/10.5061/dryad.2rd9406.
