# Supplementary material for: Refuges from fire maintain pollinator–plant interaction networks
Source: Ecol Evol. 2019 Apr 30;9(10):5777–86. doi: 10.1002/ece3.5161 (PMC6540659; doi:10.1002/ece3.5161)
Supplement: Supplementary file 1 [file ECE3-9-5777-s001.docx]

Appendix S1: List of flower-visiting insect species

| Taxa | Family | Genus | Species/Morphospecies |
| --- | --- | --- | --- |
| Bee | Halictidae | *Halictus* | sp.1 |
| Bee | Halictidae | *Halictus* | sp.2 |
| Bee | Halictidae | *Halictus* | sp.3 |
| Bee | Halictidae | *Halictus* | sp.4 |
| Bee | Halictidae | *Halictus* | sp.5 |
| Bee | Halictidae | *Halictus* | sp.6 |
| Bee | Halictidae | *Halictus* | sp.7 |
| Bee | Halictidae | *Lassioglosum* | sp.1 |
| Bee | Halictidae | *Lassioglosum* | sp.2 |
| Bee | Colletidae | *Colletes* | sp.1 |
| Bee | Colletidae | *Colletes* | sp.2 |
| Bee | Ceratinidae | Ceratinidae | sp. |
| Bee | Megachilidae | *Megachile* | sp. |
| Bee | Anthophoridae | *Anthophora* | sp.1 |
| Bee | Anthophoridae | *Anthophora* | sp.2 |
| Bee | Anthophoridae | *Amegilla* | sp. |
| Bee | Apidae | *Apis* | *melifera* |
| Bee | Apidae | Apidae | sp.1 |
| Bee | Apidae | *Xylocopa* | sp.1 |
| Bee | Apidae | *Xylocopa* | sp.2 |
| Bee | Apidae | *Xylocopa* | sp.3 |
| Bee | Apidae | *Xylocopa* | sp.4 |
| Bee | Apidae | *Xylocopa* | sp.5 |
| Bee | Apidae | *Xylocopa* | sp.6 |
| Beetle | Cerambycidae | Cerambycidae | sp.1 |
| Beetle | Cerambycidae | Cerambycidae | sp.2 |
| Beetle | Cerambycidae | Cerambycidae | sp.3 |
| Beetle | Cerambycidae | Cerambycidae | sp.4 |
| Beetle | Cerambycidae | Cerambycidae | sp.5 |
| Beetle | Cerambycidae | Cerambycidae | sp.6 |
| Beetle | Cerambycidae | *Typocerus* | sp. |
| Beetle | Scarabaeidae | *Hedybius* | sp. |
| Beetle | Scarabaeidae | *Peritrichia* | sp.1 |
| Beetle | Scarabaeidae | *Peritrichia* | sp.2 |
| Beetle | Scarabaeidae | *Peritrichia* | sp.3 |
| Beetle | Scarabaeidae | *Clania* | *glenlyonensis* |
| Beetle | Scarabaeidae | *Anisomyx* | *ursus* |
| Beetle | Scarabaeidae | Scarabaeidae | sp.1 |
| Beetle | Scarabaeidae | Scarabaeidae | sp.2 |
| Beetle | Scarabaeidae | Scarabaeidae | sp.3 |
| Beetle | Scarabaeidae | Scarabaeidae | sp.4 |
| Beetle | Scarabaeidae | Scarabaeidae | sp.5 |
| Beetle | Coccinelidae | Coccinelidae | sp. |
| Beetle | Meloidae | Meloinae | sp. |
| Fly | Drosophilidae | *Drosophila* | *melanogaster* |
| Fly | Tephritidae | Tephritidae | sp. |
| Fly | Muscidae | *Ophyra* | sp. |
| Fly | Muscidae | Muscidae | sp.1 |
| Fly | Muscidae | Muscidae | sp.2 |
| Fly | Syrphidae | Syrphidae | sp.1 |
| Fly | Syrphidae | Syrphidae | sp.2 |
| Fly | Tabanidae | Tabanidae | sp. |
| Fly | Bombylidae | Bombylidae | sp.1 |
| Fly | Bombylidae | Bombylidae | sp.2 |
| Fly | Bombylidae | Bombylidae | sp.3 |
| Fly | Caliphoridae | *Lucilia* | *sericata* |
| Fly | Caliphoridae | Caliphoridae | sp.1 |
| Fly | Caliphoridae | Caliphoridae | sp.2 |
| Fly | Culicidae | Culicidae | sp. |
| Fly | Asilidae | Asilidae | sp. |
| Wasp | Sphecidae | Sphecidae | sp.1 |
| Wasp | Sphecidae | Sphecidae | sp.2 |
| Wasp | Sphecidae | Sphecidae | sp.3 |
| Wasp | Vespidae | Vespidae | sp.1 |
| Wasp | Pompilidae | Pompilidae | sp. |
| Wasp | Icheumonidae | Ichneumonidae | sp.1 |
| Wasp | Icheumonidae | Ichneumonidae | sp.2 |

Appendix S2: List of plant species

| Family | Genus | Species/Morphospecies |
| --- | --- | --- |
| Asteraceae | *Heterolepsis* | *aliena* |
| Asteraceae | *Euryops* | sp.1 |
| Asteraceae | *Gerbera* | *linnaei* |
| Asteraceae | *Berkheya* | *herbacea* |
| Asteraceae | *Helichrysum* | *moeserianum* |
| Asteraceae | *Helichrysum* | sp.2 |
| Asteraceae | *Helichrysum* | sp.3 |
| Asteraceae | *Anthanasia* | *crithinifolia* |
| Asteraceae | *Senacio* | sp. |
| Asteraceae | *Artotheca* | *calendula* |
| Asteraceae | *Cotula* | *turbinata* |
| Asteraceae | *Metalasia* | *muricata* |
| Asteraceae | *Osteospermum* | sp. |
| Asteraceae | *Felicia* | sp. |
| Asteraceae | *Hymenolepsis* | *crithmoides* |
| Asteraceae | *Helichrysum* | *cymosum* |
| Asteraceae | *Osteospermum* | sp.2 |
| Asteraceae | *Ursinia* | *punctata* |
| Asteraceae | *Senacio* | sp.2 |
| Asteraceae | *Tanacetum* | sp. |
| Asteraceae | *Euryops* | sp. |
| Boraginaceae | *Lobostemon* | *dorotheae* |
| Bruniaceae | *Brunia* | *laevis* |
| Bruniaceae | *Berzelia* | *abrotanoides* |
| Campanulaceae | *Lobelia* | sp. |
| Campanulaceae | *Lobelia* | sp.2 |
| Ericaceae | *Erica* | sp.1 |
| Ericaceae | *Erica* | *labialis* |
| Fabaceae | *Acacia* | sp. |
| Fabaceae | *Podalyria* | *myrtillifolia* |
| Fabaceae | *Psorelea* | sp. |
| Fabaceae | *Aspalathus* | sp. |
| Fabaceae | *Aspalathus* | sp.2 |
| Fabaceae | *Aspalathus* | sp.3 |
| Fabaceae | *Aspalathus* | sp.4 |
| Iridaceae | *Morea* | sp. |
| Iridaceae | *Watsonia* | *laccata* |
| Iridaceae | *Morea* | sp.2 |
| Iridaceae | *Bobartia* | *indica* |
| Iridaceae | *Ixia* | *odorata* |
| Iridaceae | *Ixia* | *scillaris* |
| Iridaceae | *Dietes* | sp. |
| Iridaceae | *Morea* | sp. |
| Montiniaceae | *Montinia* | sp. |
| Oxalidaceae | *Oxalis* | *obtusa* |
| Oxalidaceae | *Oxalis* | *purpurea* |
| Oxalidaceae | *Oxalis* | *luteola* |
| Oxalidaceae | *Oxalis* | sp.3 |
| Polygalaceae | *Muraltia* | *heisteria* |
| Proteaceae | *Leucadendron* | *salignum* |
| Proteaceae | *Protea* | *laurifolia* |
| Scrophulariaceae | *Pseudoselago* | *spuria* |
| Scrophulariaceae | *Oftia* | *africana* |
| Scrophulariaceae | *Selago* | *corymbosa* |
| Rutaceae | *Coleonema* | sp. |
| Rhamnaceae | *Phylica* | *buxifolia* |
